# Cluster Sets to Prescribe Interval Resistance Training: A Potential Method to Optimise Resistance Training Safety, Feasibility and Efficacy in Cardiac Patients

**DOI:** 10.1186/s40798-023-00634-z

**Published:** 2023-09-19

**Authors:** Kimberley L. Way, Hannah J. Thomas, Lewan Parker, Andrew Maiorana, Michelle A. Keske, David Scott, Jennifer L. Reed, Jessica Tieng, Daniel Hackett, Tess Hawkins, Christopher Latella, Rachael Cordina, Derek L. Tran

**Affiliations:** 1 Institute for Physical Activity and Nutrition, School of Exercise and Nutrition Sciences, Deakin University, Geelong, VIC 3125 Australia; 2 Exercise Physiology and Cardiovascular Health Lab, Division of Cardiac Prevention and Rehabilitation, University of Ottawa Heart Institute, Ottawa, ON Canada; 3 Curtin School of Allied Health, Curtin University, Perth, WA Australia; 4 Allied Health Department, Fiona Stanley Hospital, Perth, WA Australia; 5 School of Clinical Sciences at Monash Health, Monash University, Clayton, VIC Australia; 6 School of Epidemiology and Public Health, Faculty of Medicine, University of Ottawa, Ottawa, ON Canada; 7 School of Human Kinetics, Faculty of Health Sciences, University of Ottawa, Ottawa, ON Canada; 8 Epigenetics and RNA Biology Program, Centenary Institute, Camperdown, NSW Australia; 9 Discipline of Exercise and Sports Science, Sydney School of Health Sciences, Faculty of Medicine and Health, The University of Sydney, Camperdown, Australia; 10 Concord Centre for STRONG Medicine, Concord Repatriation General Hospital, Concord West, NSW Australia; 11 School of Health and Medical Sciences, Edith Cowan University, Joondalup, WA Australia; 12 Central Clinical School, The University of Sydney School of Medicine, Camperdown, NSW 2006 Australia; 13 Department of Cardiology, Royal Prince Alfred Hospital, Camperdown, NSW Australia; 14 Charles Perkins Centre, Heart Research Institute, Camperdown, NSW Australia; 15 Human Performance Research Centre, School of Sport, Exercise and Rehabilitation, Faculty of Health, University of Technology Sydney, Moore Park, NSW Australia

**Keywords:** Cluster set, High-intensity interval resistance training, Intra-set rest, Inter-repetition rest, Exercise training, Cardiovascular disease, Cardiac rehabilitation, Coronary artery disease, Heart failure

## Abstract

The integration of resistance training for cardiac patients leads to important health outcomes that are not optimally obtained with aerobic exercise; these include an increase in muscle mass, maintenance of bone mineral density, and improvements in muscular fitness parameters. Despite the proliferation of evidence supporting resistance exercise in recent decades, the implementation of resistance training is underutilised, and prescription is often sub-optimal in cardiac patients. This is frequently associated with safety concerns and inadequate methods of practical exercise prescription. This review discusses the potential application of cluster sets to prescribe interval resistance training in cardiac populations. The addition of planned, regular passive intra-set rest periods (cluster sets) in resistance training (i.e., interval resistance training) may be a practical solution for reducing the magnitude of haemodynamic responses observed with traditional resistance training. This interval resistance training approach may be a more suitable option for cardiac patients. Additionally, many cardiac patients present with impaired exercise tolerance; this model of interval resistance training may be a more suitable option to reduce fatigue, increase patient tolerance and enhance performance to these workloads. Practical strategies to implement interval resistance training for cardiac patients are also discussed. Preliminary evidence suggests that interval resistance training may lead to safer acute haemodynamic responses in cardiac patients. Future research is needed to determine the efficacy and feasibility of interval resistance training for health outcomes in this population.

## Key points

- Resistance training is heavily underutilised in exercise programmes for cardiac patients.
- Given the low exercise tolerance and safety concerns with resistance exercise in cardiac patients, interval resistance training may be a safer and more appropriate method.
- High-intensity interval resistance training may be a safer, time-effective method to increase muscle function in cardiac patients.

## Background

People living with cardiovascular disease often present with low muscle mass [1], poor muscle pump function [2], increased adiposity, and an array of cardiovascular issues such as hypertension and poor glucose control [3]. Additionally, most adults with cardiovascular disease are older individuals who have lower bone mineral density [4] and are more susceptible to falls [5] and have a higher fracture risk [6]. Exercise training is an important therapy in the management of cardiovascular disease and associated co-morbidities [7]. While aerobic and resistance exercise training is highly recommended for cardiac patients, resistance exercise training is underutilised and often poorly prescribed.

Resistance training is a mode of exercise that involves exerting muscular force against an external load and leads to important health outcomes that are not optimally obtained with aerobic exercise—such as an increase in muscle mass, maintenance of bone mineral density, and increase in muscular fitness parameters (i.e. muscular strength, power and endurance) [8]. Despite the well-established benefits of resistance training as part of an exercise programme, there is an exceptionally low uptake, with only 10–30% of older adults meeting the resistance training guidelines of ≥ 2 days per week [9–12]. In people with cardiovascular disease, a typical 60-min cardiac rehabilitation session will consist of only 10-min resistance training [13]. This may be attributed to safety concerns associated with resistance exercise, inadequate time spent learning proper and safe resistance exercise techniques, and poor tolerance to traditional resistance exercise prescription models that typically involve multiple sets of 6–15 continuous repetitions. However, engaging in the combination of aerobic and resistance training appears to elicit the greatest improvements in cardiorespiratory fitness in people with cardiovascular disease [14–16], which may lead to greater prognostic benefits.

**Fig. 1:**
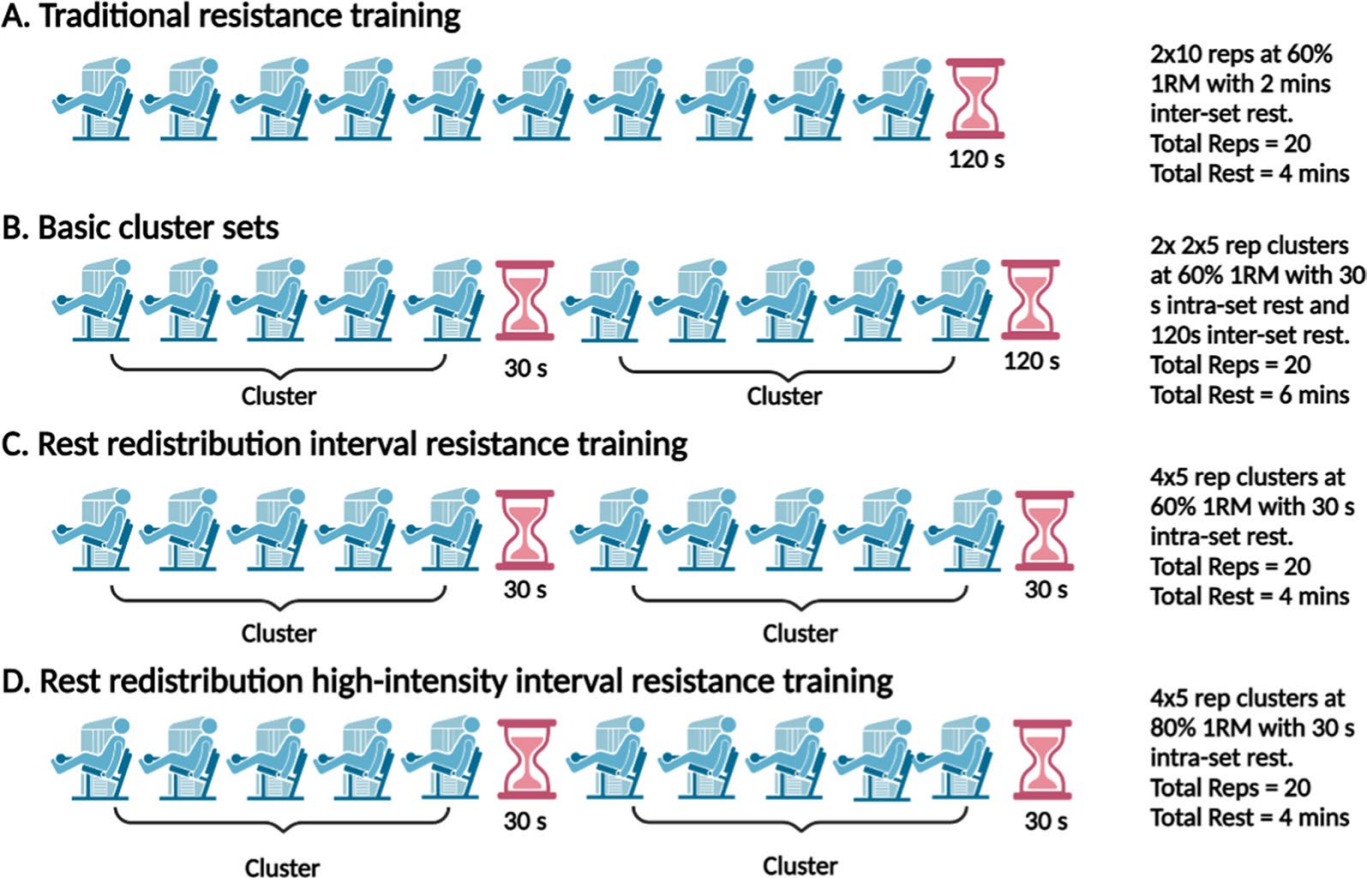
Traditional resistance training **A** compared to different interval resistance trainings based on cluster set methods (**B**–**D**). Basic cluster sets with intra-set rest are represented by Fig. 1B, inter-set rest redistribution or interval resistance training is represented by Fig. 1C and high-intensity interval resistance training by Fig. 1D

Aerobic interval training involves repeated moderate- to high-intensity bouts of aerobic activity interspersed with passive or active recovery periods [17, 18] and is often incorporated into clinical practice for cardiac patients who are severely deconditioned with low cardiorespiratory fitness to improve the patient’s tolerance to an exercise session [17]. These planned rest periods allow for the prescription and performance of higher intensities, which can reduce the volume of exercise needed to elicit health benefits. Similarly, cluster sets can be used to prescribe interval resistance training, which utilises planned regular passive rests within sets, in addition to the passive rest periods between sets found with traditional resistance training (Fig. 1). Cluster sets are a model of resistance training practice that is commonly applied in athletic populations to maximise performance and/or reduce accumulated fatigue during resistance training, but may also be an appropriate mode of resistance training for chronic disease populations including those with cardiovascular disease [19]. The rest periods between each repetition, or clusters of repetitions within a set, may reduce fatigue and the acute haemodynamic responses to resistance training and allow for the prescription and performance of higher exercise intensities to enhance muscular and health outcomes [19]. These acute responses observed with cluster sets may also improve tolerance and overall adherence to resistance exercise and allow for safer implementation of resistance training at higher intensities.

This narrative review aims to provide support for using cluster sets as a method to prescribe interval resistance training in clinical cardiac rehabilitation exercise programmes. The safety, practical application, and limitations of this resistance exercise training method will also be discussed. As this is a novel topic, a summary and critical analysis of the available research was prioritised. This did not involve a systematic search strategy, but was rather an exhaustive search conducted by the first and senior authors.

## Benefits of Resistance Training for Cardiac Patients

Common clinical presentations in cardiac patients include cardiac cachexia (up to 42% of heart failure patients) [20], skeletal muscle wasting [20–22], and peripheral muscle weakness [23, 24]; there are no pharmacological interventions available to treat such presentations; and they are not effectively addressed by aerobic training. Importantly, evidence is emerging on the prognostic benefits of engaging in resistance training [15, 25, 26].

Preserving or improving muscle function should be the primary goal when addressing skeletal muscle health in cardiac populations. Systematic reviews and meta-analyses have demonstrated the positive impact that engaging in chronic resistance training (3–26 weeks, 1–5 sessions per week including 1–12 exercises at 25–80% 1 repetition maximum [1RM] 1–10 sets of 2–30 repetitions) has for increasing muscle function (i.e. muscular strength, muscular endurance and muscular power) in patients with coronary artery disease and heart failure when compared to inactive controls [27, 28]. Hollings et al*.* demonstrated that adults with coronary artery disease improved median lower body strength by 25% (range 13–58%) and upper body strength by 46% (range 18–47%) following chronic resistance training (≥ 3 weeks of training) [27–29]. Greater muscle function improves the ability to perform activities of daily living and is strongly associated with less physical disability and continued independence [30], providing further justification for the integration of high-quality resistance exercise training for cardiac populations. Additionally, chronic resistance training improves quality of life in cardiac patients [31, 32]. To date, there are no studies that have explored the use of chronic cluster sets training in cardiac patients. The limited evidence in older healthy post-menopausal women has shown similar or superior improvements in muscle function with chronic cluster set training when compared to traditional resistance training. Ramirez-Campillo and colleagues found after 12 weeks of thrice weekly cluster set training (12 clusters of 2 repetitions with 30 s rest, 45–75% 1RM) there were significantly greater improvements in 10-m walking speed, 30-s sit to stands, timed-up and go, and quality of life when compared to the traditional resistance training group and control [33]. However, Dias et al. [34] observed similar improvements following either cluster sets or traditional resistance training in muscular strength, power, endurance and walking speed in the same population. Therefore, there is promising evidence that suggests utilising cluster sets can result in improvements in muscle function for cardiac patients.

There is limited evidence on the effect of resistance training alone on muscle mass for cardiac patients. Combined exercise training (aerobic and resistance training) approaches appear successful in improving muscle mass in patients with coronary artery disease (weighted mean difference: 0.9 kg, 95% CI 0.39–1.36 kg), albeit only three studies were pooled in this meta-analysis [35]. This is of particular importance given low muscle mass is a strong predictor of 3-year mortality in patients who have undergone a percutaneous coronary intervention [36] and all-cause mortality in individuals with and without heart failure [37]. Research in healthy older adults [38, 39], as well as other clinical population groups, such as people living with type 2 diabetes [40, 41] or overweight/obesity [42, 43], has demonstrated conflicting results regarding the efficacy of resistance training for increases in muscle mass. Cluster sets may have similar benefits as traditional resistance training for changes in muscle mass. A recent meta-analysis by Davies and colleagues demonstrated that chronic cluster set training leads to similar increases in muscle mass in young healthy adults [29]; however, the effect on cluster sets on muscle mass for older adults or clinical populations has yet to be explored. Previous studies evaluating the effect of resistance training on muscle mass are of short duration (≤ 5 months), do not implement sufficient training volume, and evaluate muscle mass using measures that may not be sensitive to small changes (i.e. dual-energy x-ray absorptiometry) [29, 44, 45]. Despite the conflicting evidence on changes in muscle mass, several studies have demonstrated improved muscle function (muscular strength [39, 40, 46–50], endurance [51–53] and power [47, 54–56]), without increases in muscle mass [46, 57, 58]; this suggests that significant neuromuscular adaptations may be facilitating improvements in muscle function. Importantly, higher muscle strength is independently associated with improved prognosis [59].

Cardiorespiratory fitness is a predictor of prognosis and survival for people with cardiovascular disease [60]. It is therefore pertinent to consider how resistance training may impact changes in cardiorespiratory fitness. Meta-analyses have shown that resistance training can improve cardiorespiratory fitness in patients with coronary artery disease and heart failure [28, 32, 61]. Hollings et al*.* reported in one of their sub-analyses (*n* = 4 studies) that cardiorespiratory fitness improved by 15.6% (95% CI 2.4 to 33.3%) following resistance training (40–80% 1RM) and may elicit similar changes to those observed after aerobic training (20.1%, 95% CI 8.3 to 34.3%) in adults with coronary artery disease [27]. However, combined resistance and aerobic training, when compared to aerobic training alone, led to near-significantly greater improvements in cardiorespiratory fitness (*n* = 12 studies, 18.4%, 95% CI 2.0 to 41.9% vs. 15.4%, 95% CI − 5.5 to 34.3%, *p* = 0.08) [27], suggesting resistance training should be incorporated into the exercise programmes of people with cardiovascular disease.

**Fig. 2:**
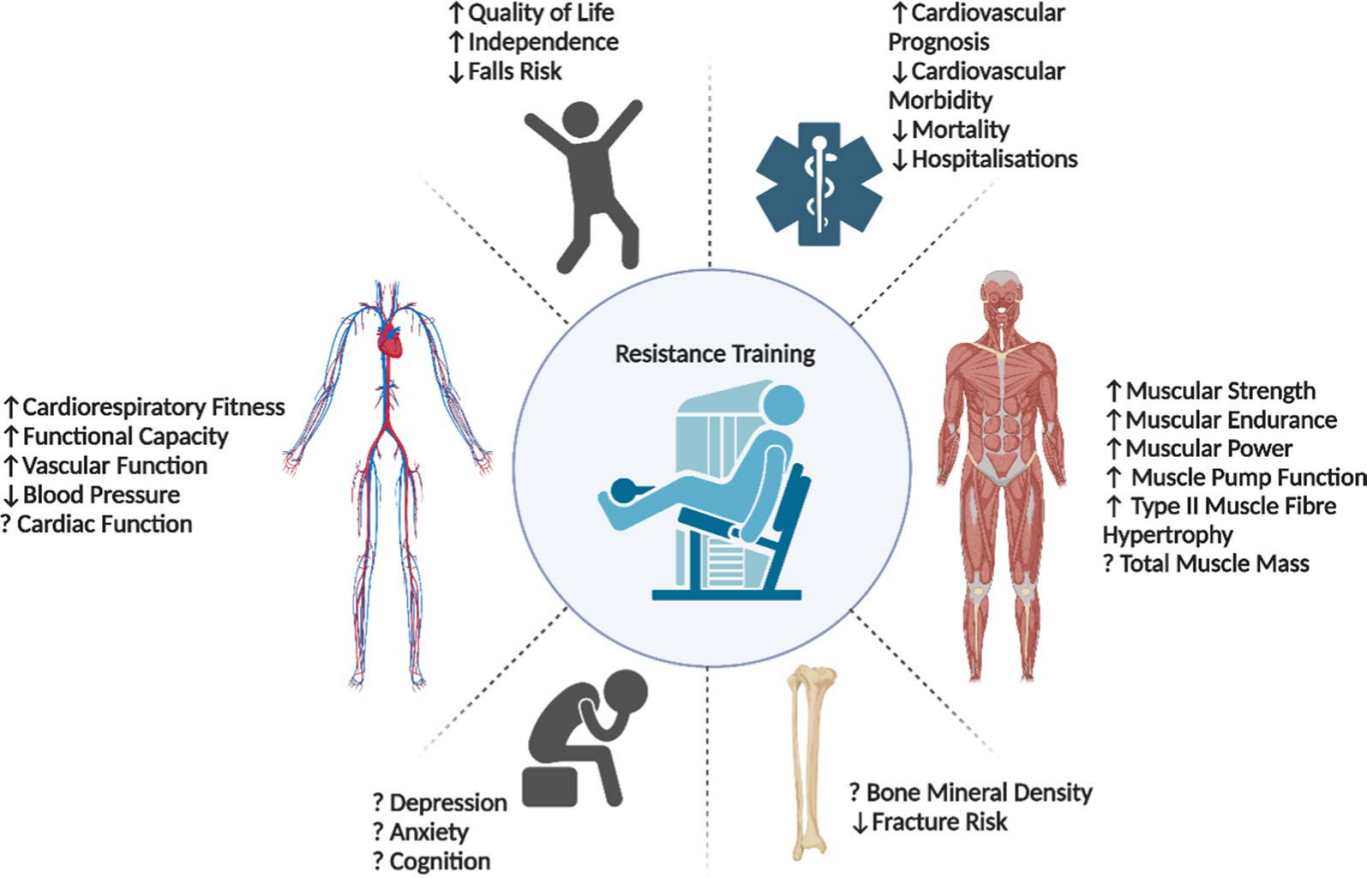
A summary of the known benefits of engaging in resistance training for cardiac patients. Possible benefits that are unclear are prefaced by a question mark

Improvements in cardiorespiratory fitness in cardiac populations following resistance training may be attributed to enhanced skeletal muscle pump function, which facilitates venous return during periods of exertion [62]. This may be particularly important in the setting of diminished preload reserve such as the Fontan circulation [63], where the augmentation of stroke volume during exercise is primarily attributed to the skeletal muscle pump [64]. De Schutter and colleagues found that in a cohort of 1171 patients with coronary artery disease, 23% did not experience an improvement in cardiorespiratory fitness with traditional cardiac rehabilitation exercise prescription consisting of 30–40 min of aerobic conditioning and light hand weights for resistance training [60]. Given these findings, a resistance training prescription with moderate-to-high loads may assist with improving cardiorespiratory fitness in low-responders to aerobic training. Such findings have been shown in a randomised crossover study in young, healthy monozygotic and dizygotic twins who completed three months of aerobic and resistance training separated by a 3-month washout period. While 86% of individuals had improved cardiorespiratory fitness with aerobic training, 10% of low responders to aerobic training were ‘rescued’ by completing resistance training, which increased cardiorespiratory fitness [65]. Additionally, previous work suggests that resistance training may also be an effective strategy for managing traditional cardiovascular disease risk factors such as poor glucose control, insulin resistance, hyperlipidaemia, hypertension and high-fat mass in adults with increased cardiovascular disease risk [66–68]; however, research has yet to comprehensively investigate the impact of resistance training on the management of cardiovascular risk factors in cardiac populations, including cluster set configurations. It is clear that resistance training may provide a plethora of health benefits for cardiac patients (Fig. 2); greater efforts to include resistance training as part of patient care are warranted.

Given that improving cardiorespiratory fitness is the primary goal for clinicians working with cardiac patients, resistance training, anecdotally, is heavily under-prescribed, with aerobic training taking precedence. Additionally, resistance training uptake is poor. In a retrospective analysis of a 12-month home-based cardiac rehabilitation programme (*n* = 358), 50% of patients discontinued the resistance training programme. Participants reported they “lacked motivation”, did not have “enough time”, were “too fatigued” and found resistance training “boring” [69]. Cluster sets may offer a method of resistance training that is more appealing and tolerable for cardiac patients. Previous studies in older adults have implemented that cluster sets have reported that patients experience less fatigue and a lower perceived exertion, which is a preferred and more enjoyable option of resistance training [70–72]. Therefore, cluster sets may be a method, which may improve adherence and compliance to resistance training in cardiac patients; however, further investigation is required.

Limited evidence indicates that high-intensity resistance training may be an effective method for improving muscle function in cardiac patients. A 12-week randomised controlled trial of twice weekly high-intensity resistance training (80% 1RM) in cardiac outpatients demonstrated significant improvements in muscular strength (+ 90%), time to exhaustion (+ 39%), body composition (-2.8% body fat and + 1.5 kg lean tissue), quality of life and mental health (depression and mood disturbances) compared to the control [73, 74]. No serious adverse events were reported. Similar findings in muscular strength were observed in a 6-month, thrice weekly, high-intensity resistance programme in older cardiac female patients; albeit no changes in body composition were found. Interestingly, none of these studies explored the effect of high-intensity resistance training on cardiovascular disease risk factors. In other populations with high cardiovascular disease risk such as adults living with type 2 diabetes, prescription of high-intensity resistance training can manage common cardiovascular disease risk factors that present in cardiac patients such as hyperglycaemia, hyperlipidaemia and high body fat [75, 76]. Cluster sets may be an appropriate method to integrate high-intensity resistance training for cardiac patients; this will be discussed further in another section.

## Safety Concerns with Resistance Exercise in Cardiac Cohorts

Although resistance training is a Class I Level A recommendation for cardiac patients[77] with clear benefits, aerobic training continues to be the dominant feature of cardiac rehabilitation. Indeed, recommendations for resistance training are often poorly defined in guidelines and resistance exercise accounts for less than one-third of a typical cardiac rehabilitation session [13]. The sub-optimal recommendations and implementation of resistance exercise in cardiac rehabilitation programmes may be attributed to safety concerns associated with resistance exercise training.

Historically, resistance training, particularly high-intensity resistance training (≥ 70% 1RM) [7], has been viewed as a potentially unsafe exercise modality for cardiac patients. This has been attributed to the notion that acute resistance exercise leads to large haemodynamic responses (increased blood pressure and heart rate), which may increase the risk of an adverse event or chronically increase afterload leading to adverse cardiac remodelling. Theoretically, the acute, transient, and repetitive increases in blood pressure may lead to myocardial ischaemia, accelerate aortic dilatation, or induce aortic dissection. These beliefs may be partially explained by early findings in this area. Notably, MacDougal et al*.* [78] reported peak blood pressure in one young healthy subject during a double leg press performed using a high relative load (> 80% 1RM to failure) to exceed 480/350 mm Hg, which led to long-standing concerns over resistance exercise prescription in patients with cardiovascular disease. While this acute increase in blood pressure is concerning, it is unlikely that a comparatively high relative load (i.e., > 80% 1RM and repetitions performed to failure) will be used in clinical practice for cardiac patients. Indeed, the recommended resistance training load for outpatient cardiac rehabilitation is 40% to 60% 1RM [8], with some guidelines progressing the load up to 80% 1RM [79]. Importantly, Haslam et al*.* reported that blood pressure responses to resistance exercise in patients with coronary artery disease were within an acceptable range when performed at a load between 40 and 60% 1RM [80].

In patients with exercise-induced pulmonary arterial hypertension, haemodynamic responses to resistance or aerobic exercise were similar during submaximal workloads (40% or 60% 1RM/peak oxygen uptake [VO_2peak_]); however, during maximal workloads (100% 1RM/VO_2peak_), haemodynamic responses were significantly lower during resistance exercise compared to aerobic exercise [81]. These findings are consistent in heart failure patients and suggest the acute haemodynamic risk associated with resistance exercise prescription is similar (or may even be lower) to aerobic exercise of similar intensity [82, 83]. Importantly, there does not appear to be deterioration of ventricular function with resistance exercise [84], and recent preliminary evidence does not support the notion that dynamic or isometric exercises accelerate increases in aortic diameter [85]. Nevertheless, isolated resistance training studies remain scarce. This makes the ‘true’ efficacy and safety of resistance exercise training challenging to interpret in clinical cohorts. Similar to high-intensity aerobic training, although adverse events during resistance training in moderate-to-high risk patients are rare, the potential risks of engaging in higher-intensity aerobic or resistance exercise should be considered by clinicians and exercise professionals.

## Exercise Prescription Models of Interval Resistance Training

While several exercise prescription models have been explored with aerobic training in clinical settings (e.g., moderate-intensity continuous training, aerobic interval training, high-intensity interval training and sprint interval training), traditional resistance training programmes are predominately applied without substantial manipulation of training principles (i.e., frequency, sets, repetitions, rest, and load). Similar to aerobic interval training, by manipulating set structures and incorporating passive rest in between efforts or ‘repetitions’ within a set (i.e., a cluster set), interval resistance training can be applied in exercise programmes. Additionally, manipulating load further diversifies the model and introduces the concept of high-intensity resistance training (loads prescribed at ≥ 70% 1RM) [7].

We propose two models of interval resistance training that may be practical and safe options for cardiac patients: (i) basic cluster sets and (ii) the rest redistribution method (Fig. 1) [86]. A basic cluster set interval resistance training approach integrates the use of short intra-set rest intervals (i.e., following a ‘cluster’ of repetitions) in addition to a longer inter-set rest period. For example, a traditional resistance training programme of 2 sets of 10 repetitions at 60% 1RM with 2-min inter-set rest could be split into 2 sets of 2 × 5 clusters with 30-s rest (after every 5^th^ repetition) and 2 min of inter-set rest (Fig. 1B). The rest redistribution approach involves dividing the total duration of the long inter-set rest for a given exercise into shorter, more frequent inter-set rests so that the total time spent resting is still equated, and the same number of total repetitions for that exercise are still completed. Using the previous example, the long 2-min rest period can be distributed evenly throughout the number of prescribed clusters—e.g. 30-s rest after every cluster of 5 repetitions until 20 repetitions are completed in this example (Fig. 1C). Each model can also be manipulated to re-distribute rest evenly after each repetition (inter-repetition rest method) and is common with this approach. These models may prove to be appropriate alternatives to traditional resistance training for cardiac populations given the exercise intolerance experienced by many patients (Fig. 1).

In low-risk, stable cardiac patients and those patients moving into the maintenance phase of cardiac rehabilitation (i.e. exercising within the community setting), utilising cluster sets may allow for higher loads (> 70% 1RM) to be well-tolerated and reduce exercise intensity and the transient haemodynamic response during resistance exercise. This can be done by prescribing cluster sets far from muscular failure (i.e., high number of repetitions in reserve). For example, cluster sets could be prescribed as 12 cluster sets of 2 repetitions at 80% 1RM with 30-s inter-set rest or as 6 cluster sets of 4 repetitions with 30-s inter-set rest. In the first prescribed example, the intensity is reduced as a lower neuromuscular response is required to perform the exercise and can minimise the duration/occurrence of Valsalva manoeuvres with more frequent rest periods. As such, cluster sets may provide a more appropriate model for high-load resistance training (i.e. high-intensity interval resistance training) in low-risk cardiac patients and those in the maintenance phase of cardiac rehabilitation (Fig. 1D).

While cluster sets may be currently employed serendipitously in clinical settings, providing these models may give practitioners a framework to improve the quality of exercise prescription of resistance training for cardiac patients and more specifically interval resistance training.

## The Benefits, Utility and Limitations of Interval Resistance Training for Cardiac Patients

When compared to traditional resistance training, the inclusion of additional intra-set, inter-repetition or distributed rest periods in interval resistance training may mitigate fatigue, improve the patient’s perception of resistance exercise and importantly reduce the haemodynamic response and cardiac load [19]. Such benefits are particularly important for cardiac patients as many present with poor exercise tolerance, low task-self efficacy regarding resistance exercise [87], and in some cases possible hypertensive responses to exercise.

### Haemodynamic Response and Cardiac Load

**Fig. 3:**
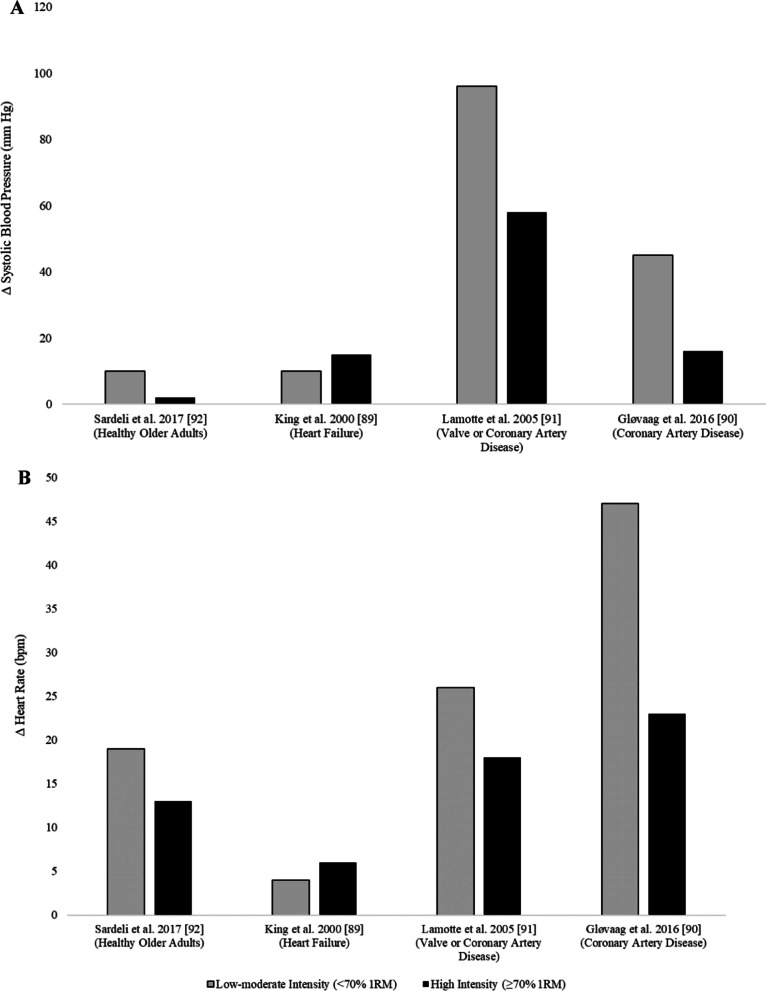
Mean change in **A** systolic blood pressure and **B** heart rate across different cardiovascular populations and healthy older adults

Recent evidence has suggested that longer duration sets occurring with higher repetition ranges, rather than load, may be the training factor leading to greater haemodynamic responses in resistance training (Fig. 3) [88–92]. A systematic review (n = 6 studies; 90 participants: 27 females, 49 males, 14 sex was not reported) conducted by Hansen et al*.* found that five of the six studies (n = 3 studies in cardiovascular disease populations; 39 participants: 4 females, 11 males, 24 sex was not reported) [90–93] demonstrated that increases in systolic blood pressure were more pronounced following sets of low-load, high repetition resistance exercise (≤ 50% 1RM, ≥ 11 repetitions) than high-load, low repetition resistance exercise (≥ 70% 1RM, ≤ 10 repetitions) even when performed to failure [90, 93] (peak systolic blood pressure: low-load, high repetition exercise: 176–220 mmHg vs high-load, low repetition exercise: 147–185 mmHg) [90, 91]. Similar haemodynamic responses were also observed in diastolic blood pressure [90, 91, 93]. These findings are consistent with Ribeiro-Torres et al*.* who showed that patients with coronary artery disease performing high-intensity interval resistance training (1 × 24 repetitions at 8RM with 15-s inter-repetition rest) had significantly lower rate pressure product (i.e. significantly lower heart rate and systolic blood pressure) than when completing traditional resistance training (3 × 8 repetitions at 8RM with 3-min inter-set rest) [94]. These preliminary findings suggest that an interval resistance training approach may result in a lower, or comparable, haemodynamic response and cardiac load (i.e., myocardial oxygen demand) in people with cardiovascular disease by reducing repetition ranges, incorporating more frequent rests and reducing exercise density (Fig. 3). Future research should focus on the haemodynamic responses and cardiac load during interval resistance exercise to determine acute responses.

### Cardiorespiratory Fitness, Musculoskeletal Fitness and Functional Capacity

To date, only one study has explored interval resistance training over several weeks in a cardiovascular disease population. In Giuliano’s pilot study, the early implementation of interval resistance training during the initial 4 weeks of an 8-week exercise programme for heart failure patients with a reduced ejection fraction was found to be superior to combined exercise training for increases in VO_2peak_ (2.4 mL/kg/min vs. 0.2 mL/kg/min) [95]. Patients in the interval resistance training group completed as many repetitions at 40% 1RM as possible in 5 min, with the individual being able to choose when to have an intra-set rest (for a minimum of 30 s).

The efficacy of traditional resistance training for increasing muscle function is widely documented in young and older healthy adults and cardiovascular disease populations. However, it is important to evaluate whether interval resistance training may be a viable method to improve muscle function for cardiac patients to mitigate reductions in muscular strength, especially with increasing age (1–2% per year from the age of 50 years) [96]. We recently demonstrated in a meta-analysis that there is no difference in muscular endurance, strength, power, or hypertrophy between traditional sets and interval (i.e., cluster) sets in apparently healthy and athletic populations [29]. Latella et al*.* also highlighted the emerging evidence of an interval resistance training approach in older adults, as well as other clinical populations such as those with neuromuscular diseases, neurological injury and pulmonary diseases. The authors postulate that interval resistance training in such populations may facilitate improvements in muscular strength and functional capacity/strength similar to those seen with traditional resistance training, but with greater tolerance and lower perceived effort [19]. Therefore, research in healthy and clinical populations suggests that interval resistance training can be a suitable alternative resistance exercise prescription to increase muscle mass and improve muscular fitness parameters. Based on these findings, interval resistance training may allow for higher-quality repetitions (e.g., better movement quality/range of motion, greater movement velocities and power output) due to less fatigue. This may be particularly important in cardiac patients given the low exercise tolerance and peripheral muscle weakness that may hinder performance using traditional resistance training methods.

These findings suggest that improvements in muscular strength are not dependent on the magnitude of accumulated fatigue during resistance training [29]. Further, interval resistance training may be a more suitable exercise prescription alternative, compared to traditional resistance training, as implementation may reduce the perception of effort [19], exacerbation of symptoms such as dyspnoea [19], and cardiovascular load. Alternatively, the prescription of higher loads, due to more frequent rest periods, can increase the total ‘volume-load’ of work performed to potentially facilitate greater adaptations in cardiac patients. The efficacy of higher loads and lower repetitions also appears to be superior compared to lower loads and high repetitions when resistance training volumes are matched. Indeed, in a combined aerobic exercise training programme with volume-matched resistance training prescription, Kambic et al*.* showed that high-load and low repetition resistance training (3 sets, 6–8 repetitions, 70–80% 1RM) was more effective at improving muscular strength and cardiorespiratory fitness compared to low-load and high repetition resistance training (3 sets, 12–22 repetitions, 35–40% 1RM) in coronary artery disease patients [97].

### Practical Limitations to Interval Resistance Training and High-Intensity Interval Resistance Training

A key limitation of interval resistance training is the additional time required to complete the exercise training session if a basic cluster interval resistance training model is applied, particularly as many international cardiac rehabilitation guidelines suggest exercise programmes include 6–8 resistance exercises [98]. Some investigators have implemented a rest redistribution model approach that can be considered synonymous with aerobic interval training and can be completed in a comparable duration to that of traditional resistance training set configurations. High-intensity interval resistance training may also be an option to reduce the duration of the session but achieve the same volume-load, as the total repetitions required to achieve a comparable volume-load are less. Therefore, if resistance exercise sessions are limited by time constraints, as is common in cardiac rehabilitation, then a rest redistribution model or high-intensity interval resistance training may be more suitable than basic cluster sets. While cluster sets are more widely recognised and used in athletic populations, the lack of knowledge of cluster sets/interval resistance training in exercise professionals working with people with cardiovascular disease may require professional development and training. As cardiac rehabilitation utilises group classes, the integration of high-intensity interval resistance training may be limited by the equipment available in the respective facility. This could be mitigated by the use of a circuit style resistance exercise class, where patients alternate between exercises, which can allow for more effective use of limited resistance training equipment (e.g. resistance exercise machines).

## Clinical Considerations and Recommendations

First and foremost, it is important for exercise professionals to instruct cardiac patients on correct exercise technique and encourage free breathing or establish breathing patterns (such as inhaling during the eccentric component and exhaling on the concentric component of the exercise) when performing resistance exercise. This will reduce the risk of musculoskeletal injuries and attenuate non-desirable acute haemodynamic responses to help ensure the patient is safe.

Further, clinicians must monitor for possible adverse responses that may occur during resistance training. Specifically, when high loads are used, there is a greater risk of a patient performing the Valsalva manoeuvre; this is difficult to avoid at loads ≥ 80% maximum voluntary contraction. This increases intrathoracic pressure and can impede venous return during exercise [99]. The significant reduction in cardiac output can result in syncope or dizziness [99]. Additionally, a secondary overshoot in systolic blood pressure following the Valsalva manoeuvre may occur with elevated intrathoracic pressure and can increase the risk of Valsalva retinopathy [100, 101], particularly in the presence of poor vascular health of cardiac patients [99]. The Valsalva manoeuvre can be avoided by instructing the patient to complete a forceful exhalation during the exercise, which will reduce the risk of such adverse responses/events.

Despite growing evidence on the importance of resistance training to counteract myopenia, increase muscular and cardiorespiratory fitness and improve functional outcomes, the majority of evidence is still obtained from aerobic exercise training—which in many cardiac cohorts has produced overwhelmingly clear evidence of benefit [102]. Interval resistance training is a technique that may alleviate some of the traditional safety concerns associated with resistance exercise and encourage the development of future research studies. While preliminary observations suggest higher loads with lower repetitions may be safer from a haemodynamic standpoint, this approach has not been well validated in cardiac populations. Furthermore, most studies investigating interval resistance training did not collect continuous measures of blood pressure during resistance exercise and blood pressure measures were taken immediately following a repetition [94]. Therefore, peak blood pressure values and mean blood pressure changes may be underestimated [98].

However, in low- to moderate-risk patients, it would be reasonable to implement interval resistance training for people with cardiovascular disease, provided the load remains the same or is only modestly higher than what is recommended in guidelines using traditional set structures [66, 103–106]. This interval resistance training approach appears to result in a lower acute haemodynamic load, which may make it a safer method of prescribing resistance exercise from a cardiac perspective. In addition, an interval resistance training configuration is likely better tolerated, would result in a better exercise performance, lower perceived effort, and in turn, would benefit adherence, compliance and health outcomes.

In a selected subset of low-risk patients with higher levels of baseline fitness and a previous history of resistance exercise training, high-intensity interval resistance training may be trialled. However, similar to all types of exercise or physical activity participation, patients should engage in a period of low-to-moderate-intensity exercise and progressively transition (ideally over 2–3 months) to higher intensities to attenuate the risk of musculoskeletal injuries or adverse cardiac events [7, 107]. Future studies should investigate the efficacy and safety of interval resistance training via cluster sets to prescribe moderate-to-high-intensity interval resistance training compared to traditional set structures in cardiac patients. Future research should address: (i) evaluating the uptake, adherence, acceptability and tolerance of interval resistance training in this population; (ii) the impact on functional and strength assessments such as the 6-min walk test sit-to-stand and hand-grip strength tests that are widely used in cardiac rehabilitation programmes; (iii) the efficacy of interval resistance training on cardiovascular disease risk factors (both physical and mental health) and body composition; (iv) continuous measurement of haemodynamics and cardiac load during interval resistance training; and v) greater inclusion of female participants to determine sex-specific responses to interval resistance training.

## Conclusion

Interval resistance training is a promising rehabilitation method for cardiac patients. Implementing cluster sets (intra-set rest periods or rest redistribution technique) to prescribe interval resistance training appears to reduce haemodynamic load, neuromuscular fatigue and perceived effort, which may optimise resistance exercise prescription and adherence. If applied in people with cardiovascular disease, the relative load used should be consistent with current guideline recommendations until further evidence is available.

## Data Availability

Not applicable.

## Abbreviations

1RM: One repetition maximum
VO_2peak_: Peak oxygen uptake
